# Ophthalmological Anatomy, with Some Illustrative Cases

**Published:** 1905-03

**Authors:** 


**Ophthalmological Anatomy, with some Illustrative Cases.** By J. HERBERT FISHER, M.B., B.S. Lond., F.R.C.S. Pp. viii., 188. London: Hodder & Stoughton. 1904.

The author states in his preface that this book has been written with the object of supplying to those engaged in ophthalmic practice a concise account in convenient form of the anatomy of the organs of vision. He has also included a number of cases illustrative of the ocular symptoms that occur in various diseases of the nervous system, in lesions of the oculo-motor nerves, in cellulitis of the orbit, and in septic thrombosis of the cavernous sinus, and these cases constitute nearly one-half of the work. They are well described and selected, and should be read with the corresponding part of the anatomical section of the book. This is especially necessary in the case of the cervical sympathetic, for we are left to infer the symptoms of a lesion here or to work them out from the cases given.

The author has not confined himself to descriptive anatomy, but quite rightly has also dealt with the functions of the structures with which he is concerned. In some of his descriptions of the anatomy of nerve centres and the course of fibres the matter is left too open—for instance, in the case of the course of the different fibres for reflex contraction of the pupils—whilst in others the statements are rather too dogmatic for the known facts. These small blemishes might easily be corrected in the next edition. We think that one or two more figures would be of advantage in helping the reader to understand the course of the fibres from the optic tracts to the cortex, the description of which is difficult. The account of the visual word centres and visual memory is too short, and requires to be considerably expanded to give it the value which we expect it to have in a special treatise of this kind.

The author does not accept the current view as to the function of the capsule of Tenon, but regards "the globe and capsule as moving harmoniously together upon the retro-ocular tissues—*i.e.* the fat packing the orbit"—and with respect to the movements of the eyeballs, he brings forward good evidence in favour of the "combined action of every extra-ocular muscle in effecting even the most simple movement."

On the whole the book is a good one, and will be useful for the purpose for which it was written; with some additions it might be made still better. In conclusion, one must commend the excellence of the type, the lightness of the volume, and last, but not least in a work of this kind, the full index.

